# Improving Dementia Care for Native American Elders: Evidence for Culturally Grounded Screening, Prevention, and Caregiving Interventions

**DOI:** 10.7759/cureus.102574

**Published:** 2026-01-29

**Authors:** Abimbola O Kolawole, Temidun O Kolawole, Barry Kissoondial

**Affiliations:** 1 College of Medicine, Central Michigan University, Mount Pleasant, USA; 2 College of Medicine, Oakland University William Beaumont School of Medicine, Rochester, USA; 3 Family Medicine, College of Medicine Affiliated Community Clinic, Central Michigan University, Mount Pleasant, USA

**Keywords:** cognitive impairment prevention, culturally grounded care, dementia screening, indigenous elders, native american health

## Abstract

Dementia is a growing health concern among Native American elders, who face a high burden of chronic disease, socioeconomic hardship, and historical trauma. Many individuals experience delayed or missed diagnoses due to structural barriers, limited specialist access, mistrust of healthcare systems, and the absence of culturally appropriate cognitive screening tools.

This narrative review summarizes current understanding of dementia prevalence, risk factors, and diagnostic challenges in Native American communities. Chronic conditions such as diabetes, hypertension, obesity, and stroke may interact with psychosocial and historical factors in ways that influence cognitive decline. Standard cognitive assessments, including the Mini-Mental State Examination (MMSE) and the clock-drawing test, may not accurately reflect cognitive function because of cultural and linguistic differences. Promising approaches include community health representative-led screening, culturally adapted assessments, community wellness initiatives, caregiver training grounded in Indigenous values, and the incorporation of traditional healing practices. Strengths-based strategies that support cultural identity and social connection may also enhance engagement in care.

Significant gaps remain, including the lack of culturally validated screening tools, limited epidemiologic data, under-resourced tribal health systems, workforce limitations, and inadequate telehealth infrastructure. Efforts to improve dementia care will require coordinated policy support, investment, and tribally led research. Culturally grounded, community-driven models may strengthen early detection, care coordination, and overall quality of life for Native American elders.

## Introduction and background

Native American elders represent one of the fastest-growing aging populations in the United States, in part due to improvements in chronic disease management, public health initiatives, and tribal healthcare infrastructure. As longevity increases, age-associated conditions such as dementia are becoming more prevalent, presenting unique clinical and public health challenges for tribal communities. Evidence indicates that Native American elders may have a higher prevalence of dementia and mild cognitive impairment than previously documented, driven by complex interactions among biological, social, and historical determinants of health [1,2]. Chronic conditions, including diabetes, hypertension, stroke, and obesity, are disproportionately prevalent in many Native communities and are well-established risk factors for cognitive decline [3,4].

Historical trauma, adverse childhood experiences (ACEs), and long-term exposure to social and economic inequities further compound dementia risk. Research demonstrates strong associations between early-life stressors and later-life cognitive and mental health outcomes, suggesting that chronic stress may contribute to neuroinflammation and cognitive vulnerability in this population [5]. Decades of structural inequities, such as limited access to preventive health services, underfunding of the Indian Health Service (IHS), and geographic isolation, exacerbate disparities in dementia diagnosis and management [1,5,6].

Standard cognitive screening tools, including the Mini-Mental State Examination (MMSE) and the clock-drawing test, were developed for Western, English-speaking populations and often fail to accurately assess elders with diverse cultural and educational backgrounds. These instruments can result in underdiagnosis or misclassification, contributing to delayed treatment. Furthermore, caregiving responsibilities frequently fall on family members due to limited long-term care facilities and a scarcity of specialized dementia services, which places significant emotional, financial, and logistical burdens on caregivers [7,8].

Despite these challenges, tribal communities are actively developing culturally grounded interventions, including prevention programs targeting cardiometabolic risk, community-based wellness initiatives, caregiver education programs, and culturally adapted cognitive assessments. Integrating Indigenous knowledge, traditional practices, and language revitalization into dementia care not only enhances early detection and treatment but also strengthens social support and cognitive resilience [8,9]. This narrative review synthesizes current evidence on the epidemiology, risk factors, screening methods, prevention strategies, caregiving experiences, and culturally tailored interventions relevant to Native American elders, aiming to inform strategies that are both clinically effective and culturally resonant.

## Review

Methods

This narrative review summarizes current evidence on dementia among Native American elders, including epidemiology, risk factors, screening practices, prevention strategies, caregiving experiences, and culturally grounded interventions. A narrative review design was selected due to the limited number of standardized studies and the need to integrate diverse sources relevant to tribal health contexts.

Search Strategy

Literature was searched in PubMed, SCOPUS, and Google Scholar using combinations of the following terms: “Native American dementia,” “American Indian cognitive impairment,” “Indigenous caregiving,” “cognitive screening American Indian,” “tribal chronic disease prevention,” and “culturally safe dementia care” to identify relevant studies published from 2000 to 2025.

Inclusion/Exclusion Criteria

Sources were included if they (1) focused on American Indian adults living in the United States, (2) addressed dementia prevalence, cognitive assessment, chronic disease risks, caregiving, or culturally adapted intervention models, and (3) were peer-reviewed articles or reputable federal/public health reports published between 2000 and 2025. Sources were excluded if they did not include American Indian or Alaska Native adults, or if these populations were combined with others without separate analysis. Publications were also excluded if they failed to address dementia, cognitive assessment, chronic disease risks, caregiving, or intervention models relevant to cognitive health. Additionally, non-peer-reviewed materials such as blog posts, unsupported opinion pieces, editorials without relevant content, and unpublished dissertations were not considered. Studies conducted outside the United States were excluded, as were articles in which dementia or cognitive health was only briefly mentioned without substantive analysis.

Study Selection and Data Extraction

Two rounds of screening were conducted by AOK and TOK. Titles and abstracts were first reviewed for relevance, followed by full-text assessment. Data were extracted regarding study population, design, primary outcomes, cultural considerations, and relevance to dementia care. Federal reports (e.g., IHS) were evaluated for epidemiologic and health system context. Any discrepancies between reviewers during the screening or selection process were resolved through discussion and consensus.

Synthesis Approach

Because study designs and outcomes varied across sources, findings were synthesized narratively and organized into thematic areas: epidemiology, diagnostic barriers, screening approaches, prevention strategies, caregiving experiences, and emerging culturally grounded care models.

Epidemiology of dementia in Native American elders

Early research on dementia in Native American populations was sparse, but the Strong Heart Study, the largest and most comprehensive cardiovascular cohort study of American Indians, provided foundational epidemiologic insights. Among adults aged 65 years and older, investigators identified substantial prevalence rates of both dementia and mild cognitive impairment, with cognitive decline closely linked to the high burden of cardiometabolic disease present in many tribal communities [2]. Consistent with these findings, Native American populations experience disproportionately elevated rates of diabetes, hypertension, stroke, obesity, and hyperlipidemia, all of which are well-established risk factors for accelerated cognitive decline and dementia [3,4].

Beyond biomedical factors, historical and psychosocial determinants also play a critical role in shaping dementia risk. Brockie TN et al. documented strong associations between ACEs and later-life depression, post-traumatic stress disorder, and poorer physical health among American Indian adults, highlighting a plausible connection between chronic stress exposure, neuroinflammatory pathways, and long-term cognitive vulnerability [5]. These findings align with broader evidence suggesting that historical trauma, social marginalization, and cumulative stressors contribute to elevated cognitive health disparities in Indigenous communities.

National health system data reinforce these epidemiologic trends. The Indian Health Service: Trends in Indian Health report identifies persistently high chronic disease prevalence, limited access to specialized neurological and geriatric services, and ongoing resource constraints within tribal health systems, all factors that influence dementia risk, diagnosis, and long-term outcomes for Native elders [1].

Barriers to dementia diagnosis

Structural Barriers

Native American elders encounter substantial structural barriers that impede timely and accurate dementia diagnosis. Many tribal communities experience geographic isolation, limited transportation options, shortages of healthcare providers, and fragmented care coordination across local, tribal, and federal systems. Chronic underfunding of the IHS further exacerbates these challenges; per-capita IHS expenditures remain significantly lower than those of Medicare or Medicaid, constraining access to neurology, geriatrics, and memory care specialists [1,6]. As a result, dementia evaluations often fall to primary care generalists who may have limited training in cognitive assessment, increasing the likelihood of delayed detection or misdiagnosis.

Telehealth expansion has improved access to behavioral health services in several Native communities, but its application to dementia care remains inconsistent. A rapid review of telebehavioral health implementation documented substantial growth in virtual care programs; however, persistent infrastructure limitations, such as inadequate broadband coverage, outdated equipment, and limited digital literacy, continue to hinder comprehensive tele-dementia assessments, particularly in rural and remote tribal regions [10].

Cultural and Linguistic Barriers

Standardized cognitive screening tools, including the MMSE and the clock-drawing test, were developed for Western, English-speaking populations and often lack cultural validity when applied to Native elders. These instruments may undervalue culturally embedded knowledge, rely on unfamiliar tasks, or assume formal education patterns that are not reflective of many Native American experiences. For example, the clock-drawing test may incorrectly classify cognitively intact elders as impaired due to limited familiarity with analog clocks or tasks unrelated to their cultural context [11]. Such inaccuracies contribute to diagnostic uncertainty and undermine trust in the assessment process.

Historical Trauma and Mistrust

Historical trauma remains a profound barrier to dementia diagnosis in Native communities. The long legacy of forced relocation, boarding schools, cultural suppression, and systemic discrimination has contributed to widespread mistrust of federal and non-Native healthcare systems. This mistrust may manifest as reluctance to participate in cognitive screening, skepticism toward medical recommendations, or avoidance of diagnostic procedures, particularly when evaluations are conducted by non-Native clinicians [5,6]. These dynamics contribute to measurable disparities in diagnosis rates; Warren and Rohlfing reported that American Indian patients were significantly less likely than White patients to receive a dementia diagnosis, even when presenting with comparable symptoms and clinical histories [7].

Culturally grounded dementia screening approaches

Improving Instrument Validity

One of the most persistent challenges in dementia care for Native American elders is the absence of cognitive assessment tools that reflect Indigenous cultures, languages, and lived experiences. Conventional screening instruments are often culturally incongruent and may underestimate cognitive abilities. The Canadian Indigenous Cognitive Assessment (CICA) offers an instructive model for addressing this gap. Developed collaboratively with First Nations communities, the CICA incorporates culturally relevant content, including storytelling, culturally familiar visual cues, and community-informed scoring methods, and has demonstrated improved screening accuracy and acceptability among Indigenous populations [12]. Although the CICA was created in a Canadian context, its underlying principles provide a strong conceptual foundation for developing culturally safe assessment tools tailored to U.S. tribal nations.

Adapting Existing Tests

In the absence of fully validated Indigenous-specific instruments, many tribal health programs have begun modifying existing cognitive tests to enhance cultural relevance. Such adaptations may involve adjusting test language, removing items that rely on Western or urban experiences, or substituting culturally grounded objects, stories, and orientation prompts. Evidence from broader cross-cultural neuropsychology supports the effectiveness of these approaches, showing that culturally adapted assessments yield more accurate diagnostic results and are better received by communities historically underserved by mainstream health systems [11]. While not a complete solution, these interim modifications represent an important step toward reducing diagnostic bias.

Use of Community Health Representatives (CHRs)

CHRs constitute one of the most trusted and culturally attuned workforces within Native American communities. As part of the long-standing IHS Community Health Representative Program, CHRs play key roles in chronic disease management, health education, and patient navigation across diverse tribal settings [13]. Expanding their involvement in dementia screening could substantially improve early detection. CHRs are uniquely positioned to provide culturally informed explanations of cognitive tests, assist with home-based or community-based assessments, identify early signs of cognitive decline, and facilitate timely referrals to clinical providers. Their deep familiarity with tribal customs, languages, and family structures enhances both the accuracy of screening and the willingness of elders to participate in diagnostic processes.

Prevention strategies for dementia in Native American communities

Cardiometabolic Risk Reduction

Native American populations experience some of the highest rates of diabetes and other cardiometabolic conditions in the United States, including hypertension, obesity, and dyslipidemia [3]. Given the strong association between vascular disease, diabetes, and cognitive decline, interventions targeting these risk factors are critical components of dementia prevention. The Special Diabetes Program for Indians (SDPI) has demonstrated notable improvements in glycemic control, blood pressure management, and lipid profiles across multiple tribal communities [4]. Although long-term cognitive outcomes remain to be fully studied, these improvements in cardiometabolic health are likely to reduce downstream dementia risk.

Community-Based Chronic Disease Programs

Culturally tailored, community-based interventions have shown promise in reducing chronic disease burden while promoting overall well-being. Programs that integrate traditional foods, culturally relevant nutrition education, physical activity, and community gatherings not only improve cardiovascular and metabolic health but also enhance psychological resilience [4]. Early implementation of such programs in adulthood or midlife may have cascading benefits for brain health, potentially delaying the onset of dementia.

Cultural Identity, Language, and Cognitive Resilience

Engagement with Indigenous language, culture, and traditional practices may serve as protective factors for cognitive health. Research by Goss CW et al. demonstrates that participation in language revitalization programs and cultural activities supports cognitive resilience, enhances memory function, and strengthens neural pathways through increased social and cultural engagement [9]. For many Native elders, involvement in traditional ceremonies, storytelling, and land-based activities reinforces social integration, community connectedness, and cultural continuity, all of which may mitigate risk for cognitive decline.

Telehealth Expansion

Telebehavioral health programs have grown substantially in Native communities and present a promising avenue for dementia prevention. Remote interventions can provide education on lifestyle modification, facilitate caregiver support, and deliver secondary prevention programs even in geographically isolated areas [10]. When coupled with culturally sensitive approaches, telehealth has the potential to expand access to prevention resources, improve adherence to chronic disease management, and promote early intervention in at-risk elders.

Caregiving in Native American families

Cultural Significance of Caregiving

In Native communities, caregiving is deeply rooted in cultural values such as respect for elders, reciprocity, and collective responsibility. For many families, providing care is not merely a practical necessity but a continuation of traditional roles and cultural obligations, reflecting a commitment to community cohesion and intergenerational support [8]. This perspective frames caregiving as a meaningful and valued contribution rather than an imposed burden.

Caregiver Burden and Resource Gaps

Despite these cultural strengths, caregiving in Native communities often entails significant stress and logistical challenges. Many caregivers face limited respite services, scarce long-term care facilities, and few dementia-specific programs on reservations. Financial constraints, geographic isolation, and restricted access to trained specialists further exacerbate these pressures. Research by Spencer SM et al. found that Native American caregivers report higher emotional and logistical burdens than non-Native caregivers, primarily due to limited formal support networks and insufficient access to dementia-trained healthcare professionals [8]. These factors highlight the need for targeted support interventions that address both practical and psychosocial caregiver needs.

Culturally Safe Caregiver Training

Culturally grounded caregiver education programs have demonstrated promising outcomes in Indigenous communities worldwide. Studies show that caregiver training designed to align with Indigenous worldviews can enhance caregiver confidence, reduce stress, and improve dementia care competencies [14,15]. Adapting and expanding these culturally safe models for tribal communities in the United States could strengthen caregiver capacity, promote sustainable family caregiving, and improve overall quality of care for Native elders with dementia.

Promising culturally grounded care models

Community-Led Dementia Programs

Several tribal communities have developed elder memory programs that integrate traditional activities, arts, crafts, and communal storytelling to promote cognitive engagement. These initiatives not only stimulate memory and attention but also reinforce cultural identity and strengthen social connections, both of which are associated with improved cognitive outcomes among Native elders [8,9]. By embedding dementia care within familiar cultural contexts, these programs foster engagement, acceptance, and participation.

Integration of Traditional Healing

Traditional healing practices, including ceremonies, smudging, talking circles, and the use of herbal medicine, often coexist alongside biomedical approaches to dementia care. When incorporated respectfully, these practices can enhance comfort, reduce behavioral symptoms, and build trust between Native elders, their families, and healthcare providers [5,6]. Integrating Indigenous knowledge with conventional care underscores the importance of culturally safe, holistic approaches that align with community values.

CHRs as Dementia Navigators

Expanding the role of CHRs to include dementia navigation represents a promising strategy to improve continuity of care, facilitate home-based support, and enhance culturally sensitive communication between families and clinical providers [13]. Given their longstanding presence and trusted status within tribal health systems, CHRs are uniquely positioned to bridge gaps between formal healthcare services and community needs, promote early detection, and assist families in navigating complex care pathways.

Discussion

This narrative review highlights the critical need for culturally grounded approaches to dementia care among Native American elders. Evidence underscores the disproportionate burden of dementia in these populations, shaped by high cardiometabolic risk, historical trauma, and structural inequities. Addressing dementia effectively requires interventions that integrate biomedical, sociocultural, and community-driven strategies. Culturally tailored interventions across screening, prevention, caregiving, and integrated care domains are summarized in Table 1.

**Table 1:** Culturally grounded dementia screening, prevention, and caregiving interventions for Native American elders. Interventions are grouped by domain and highlight key features, evidence-based outcomes, and supporting references. CHR: Community Health Representative; SDPI: Special Diabetes Program for Indians.

Domain	Intervention / Strategy	Key Features	Evidence / Outcomes	References
Screening	Culturally Adapted Cognitive Assessments	Incorporates storytelling, traditional tasks, familiar objects, and tribal language	Improved diagnostic accuracy and community acceptability	[11,12]
	Community Health Representative (CHR)-Led Screening	Home visits, culturally safe explanations, referral support	Increased screening uptake and community trust	[13]
Prevention	Cardiometabolic Risk Reduction (SDPI)	Management of diabetes, hypertension, and lipid levels	Reduced risk factors for cognitive decline	[3,4]
	Cultural Engagement and Language Revitalization	Participation in ceremonies, storytelling, and Indigenous language use	Enhanced cognitive resilience and memory function	[9]
	Community-Based Wellness Programs	Physical activity, nutrition, and engagement in tribal arts	Reduced chronic disease burden and improved social connectedness	[4]
	Telehealth-Based Interventions	Remote education, caregiver support, and secondary prevention programs	Expanded access in rural areas; potential for early intervention	[10]
Caregiving	Culturally Safe Caregiver Training	Incorporates Indigenous values, community mentorship, and tailored education	Reduced caregiver stress and improved quality of care	[14]
	Family and Community-Based Support	Intergenerational care and reinforcement of traditional roles	Maintains cultural continuity and social support	[8]
Integrated Care Models	Tribal Memory Programs and Traditional Healing	Land-based activities, smudging, talking circles, and arts	Improved cognitive engagement, behavioral symptom management, and trust in care	[5,6,8,9]
	CHR Dementia Navigation	Care coordination, home-based visits, cultural liaison	Enhanced continuity of care and early detection	[13]

Culturally Adapted Cognitive Screening Pathways

One promising approach involves a multi-step, culturally grounded dementia screening pathway. This model incorporates CHR-led home visits, culturally adapted screening tools, warm handoffs to primary care, telegeriatric consultation when necessary, and community-based caregiver support programs. Implementation of such pathways within tribal health systems has the potential to improve early detection, enhance care coordination, and foster trust between elders, families, and healthcare providers. By embedding screening within familiar cultural and community contexts, this model addresses both logistical and sociocultural barriers that contribute to underdiagnosis [11-13].

Policy and Research Gaps

Significant policy and research gaps continue to hinder the development of effective dementia care systems in Native American communities. Foremost is the scarcity of culturally validated cognitive screening tools; few instruments have been specifically tested with Native populations, and there is an urgent need for culturally safe assessments modeled after tools such as the CICA to improve diagnostic accuracy and acceptability [12]. Reliable epidemiologic data are also limited, as many tribes experience low diagnosis rates, constrained neurologic specialty access, and reporting challenges within the IHS, underscoring the need to strengthen tribal data sovereignty and support community-led research initiatives [1,7]. Chronic underfunding further restricts tribal health systems’ ability to recruit specialists, establish memory programs, and sustain dementia services, resulting in uneven care access and quality [1,6]. Workforce development is another critical need, with limited training opportunities for CHRs, primary care clinicians, and caregivers in culturally grounded dementia care, including Indigenous caregiving practices and appropriate assessment techniques. Finally, major gaps persist in telehealth infrastructure: although telebehavioral programs continue to grow, many rural reservations face inadequate broadband access and limited digital literacy, impeding the delivery of tele-dementia assessment, education, and follow-up care [10]. Targeted investment in digital infrastructure is essential to overcoming these geographic barriers and expanding culturally appropriate dementia services to isolated Native communities.

Implications

Taken together, these findings highlight the importance of tribally led, culturally resonant approaches that combine community engagement, traditional practices, and clinical expertise. Culturally adapted screening pathways, strengthened workforce capacity, and targeted policy investments represent actionable strategies for reducing disparities in dementia detection and care. Future research should focus on validating Indigenous-specific cognitive assessments, evaluating the long-term cognitive impact of cardiometabolic and culturally based prevention programs, and assessing the effectiveness of integrated care models within diverse tribal contexts.

Strengths and Limitations

This narrative review provides a comprehensive, interdisciplinary synthesis of current evidence on dementia among Native American elders, integrating epidemiologic findings, structural and cultural factors, chronic disease influences, screening challenges, prevention initiatives, and caregiving experiences. A major strength is the inclusion of diverse data sources, peer-reviewed studies, federal health reports, and community-based research, which allows for a holistic understanding of dementia within tribal contexts. The review also centers Indigenous perspectives and highlights culturally grounded interventions, reflecting community priorities often underrepresented in mainstream dementia literature. Finally, by organizing findings across screening, prevention, caregiving, and integrated care domains, the review offers actionable, practice-oriented insights for clinicians, policymakers, and tribal health programs.

This review is limited by the small and heterogeneous evidence base on dementia in Native American communities, which reduces comparability across studies. Cultural and tribal diversity also makes it difficult for any single synthesis to reflect all community contexts. Finally, as a narrative review, the findings may be influenced by selection bias despite efforts to use broad search strategies and high-quality sources.

## Conclusions

Dementia among Native American elders represents an urgent and growing public health challenge, driven by high cardiometabolic risk, historical trauma, and longstanding structural inequities. Effective dementia care in these communities requires culturally grounded approaches that integrate traditional knowledge, language, and community leadership with clinical expertise. Promising strategies include CHR-led dementia navigation, culturally adapted cognitive screening tools, community-based prevention programs, and caregiver support initiatives that align with Indigenous worldviews. These approaches not only enhance early detection and care coordination but also foster trust, cultural continuity, and cognitive resilience among elders.

Addressing persistent gaps, such as limited culturally valid screening instruments, insufficient epidemiologic data, underfunded tribal health systems, workforce shortages, and telehealth infrastructure limitations, will require coordinated policy action, investment, and tribally led research initiatives. Collaborative partnerships between tribal communities, healthcare providers, and policymakers are essential to co-develop dementia care pathways that respect tribal sovereignty, cultural values, and lived experiences. By centering culturally resonant, community-driven models of care, it is possible to improve the quality, accessibility, and effectiveness of dementia services for Native American elders, ultimately reducing disparities and supporting healthy aging within these communities.
